# Parental Attitude Toward the Engagement in Physical Activity of Their Children with Type 1 Diabetes Mellitus in Hungary

**DOI:** 10.3390/children12050612

**Published:** 2025-05-07

**Authors:** Ildikó Balatoni

**Affiliations:** Clinical Center, University of Debrecen, H-4032 Debrecen, Hungary; balatoni@med.unideb.hu; Tel.: +36-306762214

**Keywords:** physical activity, children, type 1 diabetes mellitus, parental attitude, quality of life

## Abstract

Background/Objectives: Physical activity plays an essential role in a healthy lifestyle. For children, the development of an encouraging attitude toward exercise can define a positive life-long behaviour. Type 1 diabetes mellitus (T1DM) is a metabolic disorder that usually develops in early childhood and severely affects glucose metabolism. Associated hypo- and hyperglycaemic conditions can dramatically interfere with the patient’s everyday life. Since exercise significantly alters the glucose consumption of the body, this might influence how T1DM patients view physical activity. As parental guidance is critical in their children’s behaviour, we investigate how parents of T1DM children relate to the engagement in physical activity of their children as compared to parents of healthy children. Methods: A self-reported survey was conducted among those parents whose T1DM children were cared for at the Paediatric Clinic of the University of Debrecen, Hungary. All together, 318 children, 140 with T1DM and 178 healthy peers, participated in the study. Results: We found no significant difference in the body mass index of healthy and T1DM children and, furthermore, no significant difference was observed in HbA1c levels in exercising and non-exercising T1DM children. Nevertheless, while 67.6% of the healthy children regularly engage in physical activity, only 57.5% of T1DM children do so (*p* = 0.044). Importantly, parents whose T1DM child exercised regularly believed that daily PhysEd classes improved their children’s health and had positive effects on their attitude toward exercise. In contrast, parents of children who did not regularly exercise were significantly less convinced. Conclusions: These findings highlight the importance of targeted educational efforts to foster positive attitudes toward physical activity among families with T1DM children and contribute valuable insights into how parental perceptions may influence children’s engagement in exercise.

## 1. Introduction

Childhood plays a key role in developing our adult physical activity habits. Learning to lead a conscious lifestyle of daily physical activity is essential to becoming a healthy adult [1]. Children with type 1 diabetes (T1DM) are a special group in terms of physical activity habits, as their parents’ attitudes toward the disease and sports are also crucial [2].

Type 1 diabetes mellitus (T1DM) is the most common childhood endocrine disease, with 108,200 newly diagnosed cases under 15 years of age each year globally [3] and an increasing incidence [4,5,6]. According to the International Diabetes Federation (IDF) 2023 report, some 169,000 children and adolescents live with T1DM in the EU (14% in the world) [7]. Most children are diagnosed between the ages of 10 and 14 years, but, in recent years, the age of diagnosis has been shifting toward a younger age, with more children diagnosed between the ages of 5 and 9 years [8,9,10].

Barkai et al. analyzed, among other things, the incidence and prevalence of T1DM among children and adolescents in Hungary between 2001 and 2016 [11]. They found that the incidence and prevalence of T1DM have been steadily increasing among children and adolescents, with an annual growth rate of T1DM incidence of 2.5%/year.

T1DM children are more vulnerable than adult patients in terms of blood glucose metabolism, as they have greater glycaemic variability and are more vulnerable to parental decisions about therapy. Based on the recommendation by the treating physician, it is essential to involve parents in the therapy management of children, as T1DM requires multiple therapeutic decisions per day, and in the case of children, this should be the responsibility of the parent. At an appropriate age, therapeutic decision-making should be a joint responsibility of the patient and parents, with the child gradually becoming more and more independent [12,13].

Several studies have shown that family functioning, parental psychosocial characteristics, parental coping skills, and child characteristics interact and significantly influence the effectiveness of T1DM management [14,15,16].

The relationship between chronic disease and physical activity has a large and comprehensive international body of literature [17,18]. Although professional recommendations may vary in the amount and intensity of exercise recommended for healthy children, regular physical activity is always recommended. For the average non-T1DM child, the American Academy of Pediatrics recommends at least 60 to 90 min of moderate-to-vigorous intensity physical activity per day in toddlerhood, 90 to 120 min in preschool, and at least 60 min of moderate-to-vigorous intensity physical activity per day in school and puberty [19,20,21,22,23,24].

The recommendations are based on the insight that reduced physical activity is associated with higher body weight and higher incidence and mortality from cardiovascular disease [25].

The recommendations for the T1DM group do not differentiate between diabetic and healthy children in terms of duration and intensity of exercise, but they do emphasize the need for each T1DM child to tailor the amount and type of exercise to their own needs, creating an independent exercise plan. They also recommend a minimum of 60 min of moderate-intensity physical activity per day, at least three times per week, with a schedule that ensures that no more than 2 days go by without physical activity. However, there is unanimous agreement that regular physical activity is recommended and necessary for people with diabetes, as it contributes to a healthy metabolism [21,22,26,27].

Regular exercise is a particularly important part of diabetes management, as physical activity has been shown to have a large effect on blood glucose [28,29,30,31,32,33]. The body’s response to exercise depends on the balance between glucose efflux from the liver and glucose utilization by the muscles, such balance influenced by diet, intensity, and modality of physical activity, and the parameters of the patient being studied together with the therapy used and its effectiveness [34]. In addition, metabolic effects are also influenced by the predominant cellular metabolic process used by the body during exercise, with said process either aerobic or anaerobic, depending on the intensity of exercise and the state and workload of the athlete’s circulatory system.

Sports help to achieve and maintain the desired metabolic control of diabetes and play a role in the prevention of complications by improving blood pressure, endothelial function, and lipid profile. Regular physical activity increases cardiovascular and physical fitness and also plays a role in maintaining mental health, leading to higher self-esteem and a better quality of life [34,35,36,37,38,39,40].

Previous research has shown that children with T1DM are twice as likely as their healthy peers of a similar age to be affected by cardiovascular diseases in their lifetime [31,36,37,41,42]. For this reason, it is particularly important to use all available means to improve metabolism and to pay attention to the primary prevention of potential complications in children with T1DM.

Regular physical activity leads to better metabolic control and can reduce HbA1c, thus preventing complications [37,43,44]. A difference has also been shown in HbA1c between diabetics who exercise more and those who exercise less or not at all [43,45]. Furthermore, the higher the HbA1c of T1DM patients, the lower their exercise capacity, and even at HbA1c levels 1.5% higher than normal, a decrease in cardiovascular fitness has been observed [32,46,47].

While diabetes is not a barrier to exercise when metabolism is well-adjusted [48,49], previous studies have shown that diabetics exercise less than their healthy peers [43,50].

Research on the main impediments to physical activity in T1DM patients has found that fear of hypoglycaemia is the most common factor, together with a lack of motivation and time [29,30,51,52]. In children, parental patterns and motivation also play a role, and this is more prevalent in younger children. Parents can be a strong motivator for more physical activity. Parental motivation, sporting habits, financial resources, family logistics, and positive role models all encourage children to be more physically active. Their positive and supportive attitude toward physical activity is, therefore, essential for children to develop a healthy lifestyle. Another very important barrier to exercise in T1DM children is the fear of hypoglycaemia among the parents. This fear and barrier exist even if the child has never experienced severe hypoglycaemia [33,37,53].

As a result of the abovementioned barriers, children with diabetes do less sports than their non-diabetic peers [54], and fewer children with T1DM play competitive sports. On average, these patients spend less time exercising after diagnosis [55]. In addition, Matson et al. found that T1DM patients exercise 25% less and choose less intense exercise than their healthy peers [56].

Based on the above, our research seeks to investigate parental perceptions regarding physical activity in children with T1DM, identifying specific barriers and motivational factors, in the context of a disadvantaged region in Hungary. We hypothesize that fear of hypoglycaemia and parental concerns about symptom aggravation might act as barriers to regular physical activity in children with diabetes mellitus. We further assume that these concerns and behaviours would not differ significantly between boys and girls. Finally, we expect that the severity of the disease would be inversely related to physical activity levels, and that this would be reflected in changes in the child’s BMI.

The results of our research are gap-filling, as there is limited evidence in the international literature regarding parental attitudes toward physical activity in children with T1DM. Furthermore, no similar studies have been conducted in Hungary—particularly in the Northern Great Plain region, which is characterized by low levels of education and per capita income, along with high rates of unemployment and chronic disease prevalence [57].

## 2. Materials and Methods

### 2.1. Study Design

We examined the physical activity habits, favourite sports, and various barriers to physical activity in children with type 1 diabetes mellitus and healthy controls using a structured questionnaire for data collection. The questionnaire was developed specifically for this study and consisted of the following sections: (1) socio-demographic characteristics, (2) physical activity, and (3) quality of life (Table 1).

To assess the physical activity of the participants, we employed a short version of the International Physical Activity Questionnaire (IPAQ) [58,59] and incorporated additional specific questions on physical activity from a validated questionnaire utilized in our previous studies [60,61]. Parents were also asked about their children’s other daily activities, such as ‘Does your child attend physical education classes at school?’, ‘Does your child attend physiotherapy?’. In keeping with good quality design, the questionnaire was reviewed by two specialists.

We asked four questions about socio-demographic data, followed by eight questions related to sporting habits in the form of multiple-choice answers. The questionnaire was answered by the parents, detailing how often, what, and where the child likes to play sports. One more question was asked about the reasons for not playing sports, where several answers could be ticked. This was followed by 12 statements about the relationship between physical activity and diabetes, covering the benefits of physical activity, the appropriateness of physical activity for children with diabetes, and the parents’ feelings about their child’s exercise, with responses given on a 5-point Likert-type scale: ‘strongly disagree’, ‘disagree’, ‘no opinion’, ‘agree’, and ‘strongly agree’.

The children’s quality of life was assessed using the diabetes symptom items of the Pediatric Quality of Life Inventory 3.0 Type 1 Diabetes Module (PedsQL 3.0 DM) in the last part of the questionnaire, a validated tool designed for children with type 1 diabetes [62,63].

All questions were answered by the parents of the children in this study during their visit for a check-up. Participation in the study was voluntary and anonymous. The interviewers, who are assistants working at the investigation site, recruited parents of sick children. Upon being informed of the purpose and procedure of the study, the parents provided written consent to participate.

A test was run for the full questionnaire and the Cronbach’s alpha was found to be 0.830. To standardize the data collection process, the three interviewers underwent a training session specifically designed to align data collection procedures.

The study adhered to the Declaration of Helsinki on Ethical Principles for Medical Research Involving Human Subjects and was approved by the Medical Research Council of Hungary (ETT-TUKEB: 24634-4/2018/EKU).

### 2.2. Participants

In total, 318 children between the ages of 4 and 18 years participated in this study, divided into a clinical group (*n* = 140) of type 1 diabetes mellitus (T1DM) patients and a healthy group (*n* = 178) with similar demographic factors to the study group. The diabetic children were examined at the diabetes outpatient clinic in the Department of Pediatrics at the University of Debrecen during their regularly scheduled clinic appointments. All children who attended the diabetes outpatient clinic at the time of the survey were included in the study.

Inclusion criteria for the control group required that participants be between 4 and 18 years of age, receive the standard number of physical education (PE) lessons, attend an institution without a specialized sports profile, and have no chronic illnesses that limit their physical activity. Overweight status was not considered a medical condition; therefore, the term “healthy children” is used throughout the text when referring to members of the control group. Questionnaires for the healthy children were completed at family-oriented events. Respondents were randomly selected from among those who consented to participate.

In the survey, the proportions of boys and girls were similar in both groups: 51.8% boys and 48.2% girls in the diabetic group, and 44.9% boys and 55.1% girls in the control group. The demographic characteristics of the participants are reported in Table 2.

### 2.3. Study Variables

Socio-demographic characteristics included sex, age (4–10, 11–14, 15–18 years), education level, place of residence, and family income status. In the present study, we assessed body mass index (BMI) and physical activity (PA) based on self-reported answers. BMI was calculated as body weight in kilograms divided by height in meters squared (kg/m^2^). The BMI number was identified in the CDC’s BMI-for-age growth chart (for girls or boys) to obtain a percentile ranking. Children above the 85th percentile are considered overweight, and those below the 5th percentile are considered underweight [64,65]. In the analysis, participants were divided into three groups based on their place of residence: village, town, and the county seat Debrecen. For physical activity, patients were grouped as follows: PA1, less than three times per month; PA2, one to two times per week; PA3, three to four times per week; and PA4, more than five times per week.

The diabetes monitoring inquired whether the participant used an insulin pump or relied solely on self-injection and monitoring, as well as their family history and duration of the disease. During the visit, glycated haemoglobin (HbA1c), which is used to assess diabetes control [66], was measured, ranging from 5.7 to 10.2%. HbA1c data were derived from capillary blood samples measured by local laboratory methods using high-performance liquid chromatography.

### 2.4. Family History

Survey respondents were asked if either of their parents had diabetes and if anyone in their family had type 1 diabetes. We determined family history as a first-degree relative with diabetes (parent and/or sibling). According to our results, the vast majority of children did not have a parent with diabetes (83.6% and 88.2% for diabetic and healthy children, respectively) and did not have a family history of type 1 diabetes (72.9% and 76.3% for diabetic and healthy children, respectively).

### 2.5. Data Analysis

The completed questionnaires were processed using EvaSys 8.2. software (VSL Inc., Szentendre, Hungary; http://www.vsl.hu). Descriptive statistics were used to detail the characteristics of the groups. Data are presented as the mean (SD) and counts (relative percentages), respectively. In the case of multiple responses, frequency was analyzed using multiple response frequencies and the correlation between groups was examined using multiple response crosstabs. The normal distribution of the data was checked using the Kolmogorov–Smirnov test and the Shapiro–Wilk test. Data were analyzed using non-parametric methods because the normality tests showed that the study groups were not normally distributed. The Chi-square test or Fischer’s exact test was used for comparing proportions. The significance of differences between groups was assessed using the Mann–Whitney–Wilcoxon test. To examine the variable HbA1c, a one-way ANOVA was used to determine significant differences among groups by physical activity frequency. A *p*-value less than 0.05 was considered statistically significant. Statistical analyses were performed using SPSS (Statistical Package for the Social Sciences) version 29.0 software (SPSS Inc., Chicago, IL, USA).

## 3. Results

The mean age of the control group was 12.6 ± 2.8 years, while that of the diabetes group was 13.1 ± 3.2 years, with a median diabetes duration of 6 years (few months—15 years) and a mean HbA1c of 7.8% ± 1 (mmol/mol). When the data were examined, we did not find associations among HbA1c, gender, age, and duration. The majority of children in both groups were between 11 and 18 years of age (Table 2).

By investigating the relationship between physical activity and age groups we found that healthy children aged 11–14 years were significantly more physically active than their peers (*p* = 0.001), while we did not find a significant difference among age groups in the diabetes group. Regarding the regularity of physical activity, healthy children exercised one to two times a week, while children with diabetes exercised three to four times a week on average, but there were no significant differences among age groups in any of the samples. The majority of children in both samples participated in PE classes, but few attended physiotherapies, and there were no significant differences among age groups in either case. When looking at the correlations between the various sports and age groups, in the control group we observed that the 4–10 age group was significantly more likely to prefer swimming (*p* = 0.002), while the 11–14 age group tended to choose cycling (*p* = 0.024). Analyzing data from the diabetic group we found significant differences for basketball and football (*p* = 0.028 and *p* = 0.015, respectively). We observed that the 15–18 age group chose basketball much more often than their younger counterparts. At the same time, football was the least popular sport type in the 11–14 age group. Examining the association between reasons for physical inactivity and age groups we found that significantly more children aged 15–18 did not play sports due to lack of time (*p* = 0.007) and tiredness (*p* = 0.010) in the control group, while in the diabetes group, significantly more 11–14-year-olds failed to exercise because of their health condition (*p* = 0.037).

### 3.1. Socio-Economic Factors Influencing Sports Behavior

Among the children, 63.6% of the control group and 54.6% of the diabetes group attended elementary school. The remaining children, 36.4% in the control and 45.4% in the diabetes group, attended different types of high schools (high school, vocational high school, technical college). In the studied groups, 19% in the control group and 21.6% in the diabetes group lived in Debrecen, 58.3% and 52.2% lived in different towns, while 22.6% and 26.1% lived in villages, respectively. Analysis by residence showed no significant differences in rates of physical activity between children with diabetes and healthy children. We investigated the relationship between sport of choice and place of residence, and significant differences were observed for football (*p* = 0.017) and swimming (*p* = 0.002) among healthy children and for football (*p* = 0.045) and basketball (*p* = 0.017) among children with diabetes. In the diabetic group, there was no significant correlation between the reasons for physical inactivity, while healthy children living in the village did not consider physical activity important (*p* = 0.027). However, we observed that a large proportion of children living in the city did not engage in exercise due to lack of time and fatigue.

Regarding the income situation of families, in the control and diabetes groups, we found that 45.3% and 48.4% of the families, respectively, reported a good standard of living, including financial savings. A total of 36.6% of the control group and 37.5% of the diabetes group could cover all their expenditures without being able to save money. In addition, 9.9% and 6.3% of the subjects reported poor living circumstances and suffered from poverty in their everyday lives (for the control and diabetes group, respectively).

### 3.2. Physical and Health Status of Children

**Figure 1:**
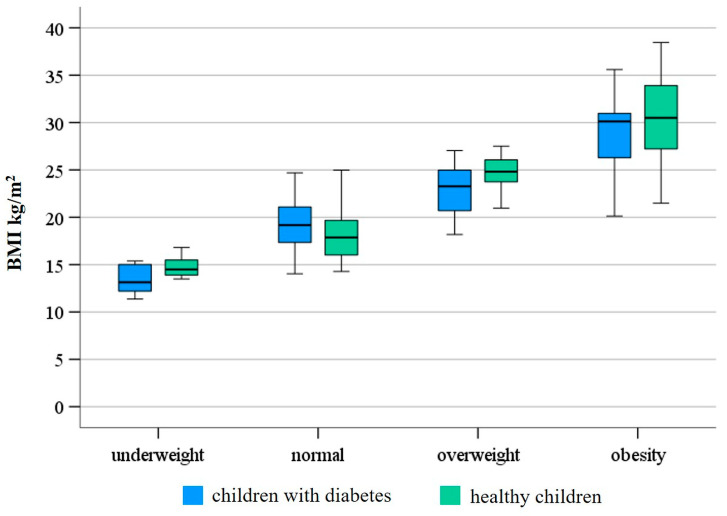
The distribution of BMI (body mass index) status of diabetic and healthy children in the survey. Note that the distribution of BMI within each weight category was similar between the T1DM and the control groups.

The BMI of participants in the control and diabetic groups varied substantially (13.49–38.45 and 11.36–38.2, respectively; minimum–maximum). Based on their BMI, 39 out of 140 children in the diabetes group were considered overweight or obese, while nine out of 140 were underweight. Among healthy children, 60 out of 178 were overweight or obese, and 13 out of 178 were underweight. The distribution of the BMI status of the diabetic and healthy children in the study is shown in Figure 1. We also assessed the correlation between children’s BMI status and their physical activity, but no significant difference was found between the groups (*p* = 0.244 for the control group and *p* = 0.827 for the diabetes group). Additionally, the examination of the BMI status also revealed that there was no significant difference between their participation rates in physical education (*p* = 0.936 for the control group and *p* = 0.791 for the diabetes group) and physiotherapy (*p* = 0.754 for the control group and *p* = 0.603 for the diabetes group). When comparing the BMI status among different sports, no significant difference was found in the diabetes group, while there was a significant difference in the control group for combat sports (*p* = 0.044) and table tennis (*p* = 0.002). We observed that diabetic and healthy children with a normal BMI chose cycling and football more frequently than their peers. Investigating the association between reasons for physical inactivity and BMI status, we observed that children with a normal BMI in both groups were significantly more likely not to exercise due to fatigue (*p* = 0.015 for the diabetes group and *p* = 0.049 for the control group). In addition, one of the main reasons for physical inactivity in obese and overweight children was a lack of time in the group of children with diabetes and a lack of motivation in the control group, according to our results.

When examining the relationship between physical activity and HbA1c levels, no significant association was found between children with diabetes who did (7.65% ± 0.96) and did not engage (7.88% ± 0.96) in physical activity.

### 3.3. Sporting Habits of Children

Looking at the proportion of regular physical activity in addition to PE classes, 67.6% (120/178) of healthy children and 57.5% (81/140) of children with diabetes engaged in some kind of exercise (*p* = 0.044). A total of 46.2% and 35.7% of control and diabetic children, respectively, exercised once or twice a week, 36.1% and 36.9% exercised three to four times a week, while 14.3% and 22.6% exercised at least five times a week (Table 2).

The most common forms of physical activities in both groups were cycling (16.9% and 25% for the control and diabetes groups, respectively) and football (18% and 15.7% for the control and diabetes groups, respectively). Furthermore, a large proportion of healthy children chose to dance (15.2%), while diabetic children chose to run (13.6%).

Children generally exercised in school as part of their afternoon activities (36.5% and 27.1% for the control and diabetes groups, respectively). Moreover, a high proportion of healthy children were active in different sports clubs (28.1%), while children with diabetes favoured outdoor activities (21.4%). Some exercised at home (11.8% and 19.3% for the control and diabetes groups, respectively).

However, even those who did not exercise regularly did frequent physical activity in both groups, such as cycling or walking. This was likely due to the fact that most of the subjects lived in small towns where cycling or walking were the most common forms of transportation. It is important to note that except for 2.9% in the healthy group and 11.7% in the diabetes group, almost all children participated in PE classes. In correlation to that, the percentage of children going to physiotherapy was very low, only 7.5% and 5.2% for the control and diabetes groups, respectively. However, the percentage of children who did not exercise due to a lack of time was very high among those who did not perform regular physical activity (12.4% and 20.7% for the control and diabetes groups, respectively). Tiredness (9% and 14.3% for the control and diabetes groups, respectively) and lack of motivation (10.1% and 10.7% for the control and diabetes groups, respectively) were the other most common motives for physical inactivity.

In the diabetes group, examining the relationship between the insulin method and physical activity, we found no significant difference between multiple daily insulin injections and pump use (*p* = 0.602). Both methods were used in similar proportions among physically active and inactive patients. We also tested the relationship between physical activity and the presence of diabetes but found no significant difference (*p* = 0.171). The vast majority of children had had diabetes for several years at the time of the study.

We also analyzed whether there was an association between diabetes in a close relative of a child with diabetes and the child’s physical activity. We found that 27.1% of children had such a relative, and the proportion of these children who regularly played sports (54.3%) was not significantly different from those without a relative with diabetes (58.5%; *p* = 0.693).

### 3.4. Parents’ Opinion on the Physical Activity of Their Children

A total of 85.9% of parents found the role of physical activity important for children with diabetes. Additionally, 77.4% answered that physical activity could improve the disease.

**Figure 2:**
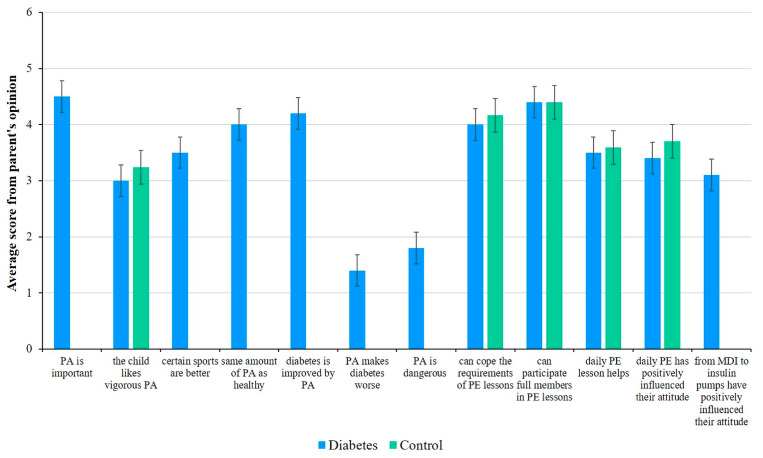
Parents’ opinions—on a scale of 1–5—on the physical activity of healthy and diabetic children. Mean ± SD of the data.

Evaluating the statements about PE classes, we found that 79.1% of parents in the control group and 74.6% of parents in the diabetes group thought that their children could meet all the requirements of PE classes. A similar proportion of parents in both groups felt that their children could participate in PE classes just like everyone else, that daily PE classes improved their child’s health, and that they had a positive effect on their attitude toward physical activity (Figure 2).

Differences in parents’ responses between those whose children exercised regularly versus those whose children did not are worth considering. In the diabetes group, parents whose children exercised regularly answered that physical activity improved their child’s diabetes (4.39; weighted average of points on a 1–5-point scale) and that their child could participate in PE classes just like healthy children (4.30). In contrast, the physically inactive group scored less positively on these items (3.82 and 3.61, respectively). The significance of everyday exercise was also different among the two groups of parents. According to the parents of children with diabetes who regularly exercised, daily PE classes improved their children’s health (3.75) and positively influenced their attitude toward physical activity (3.82). On the other hand, the parents of children who did not exercise regularly thought that the effect of daily PE classes was negligible. The perception of the importance of daily physical activity was similar among parents of healthy children. In the study groups, the statistically significant differences between children who regularly participated in physical activity and those who did not are presented in Table 3. Significant *p*-values are indicated in bold and italic, with *p* <0.05 considered statistically significant. Despite the fact that parents agreed that exercise can improve the condition of children with diabetes and disagreed that it is dangerous and worsens the condition of children with diabetes, nearly half of the children in the study did not exercise outside of PE lessons.

### 3.5. Impact of Diabetes on Children’s Quality of Life

Survey respondents reported that the most common symptoms their children had experienced in the past month were hunger (58.8%), thirst (54.5%), fatigue (59%), and low blood sugar (74.1%). Studying the association between physical activity and diabetes symptoms, we observed a significant difference only in the feeling of fatigue (Table 4). Children who did not exercise were more likely to feel tired or exhausted than their physically active peers (*p* = 0.015).

### 3.6. Gender-Based Analysis of Physical Activity of Children with Diabetes and Healthy Children

An independent analysis of the answers from girls and boys showed no significant difference in either group regarding the rate of their physical exercise, participation in PE classes, and participation in physiotherapy. For both groups, girls and boys preferred cycling. In the group of healthy children, there was a significant gender difference in this preference, with boys showing a greater interest in cycling (*p* = 0.028). In addition, girls commonly chose dancing (a significant difference observed only in the healthy children group; *p* = 0.018), while boys preferred football (a significant difference was identified in both groups; *p* < 0.001 for the control group and *p* = 0.004 for the diabetes group). We analyzed the relationship between the causes of physical inactivity and gender and found that most children did not exercise due to a lack of time, fatigue, and a lack of motivation. A significant association was only observed in lack of time (*p* = 0.039) in the group of children with diabetes.

In both study groups, the parents of girls and boys agreed that their children could cope with the requirements of PE lessons and could participate fully in group sports activities during PE classes. In the diabetic group, the parents of girls and boys agreed that physical activity was important for children with diabetes (4.51 and 4.46 for girls and boys, respectively). They also agreed that physical activity could improve their child’s health status (4.06 and 4.24 for girls and boys, respectively) and that their child could participate in the same amount of physical activity as their healthy peers (4.08 and 3.94 for girls and boys, respectively). The parents of diabetic girls were significantly less likely to agree (2.69) with the statement that their child with diabetes liked vigorous physical activity (*p* = 0.024) and were more likely to believe that certain sports were better for children with diabetes (3.68). The parents of healthy children had similar perceptions about their child’s preference for vigorous physical activity, with no significant difference found. The parents of children with diabetes disagreed that physical activity makes diabetes worse (1.45 and 1.23 for girls and boys, respectively) and that it is dangerous for children with diabetes (1.72 and 1.76 for girls and boys, respectively). The parents of boys with diabetes believed that their sons were more neutral (3.02) in their attitude toward physical activity when they switched from MDIs (multiple daily injections) to an insulin pump. Furthermore, parents thought that the introduction of daily physical education had no impact on their child’s health and attitude in the diabetic group. However, the parents of healthy boys were significantly more likely to agree with these statements (*p* = 0.026 and *p* = 0.005, respectively). Table 5 presents summaries of parents’ opinions by gender in both study groups.

**Figure 3:**
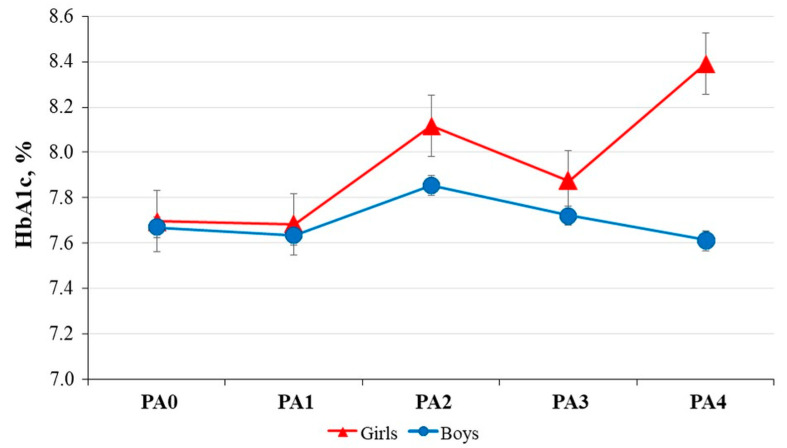
HbA1c mean values for boys and girls according to their level of physical activity. Abbreviations: HbA1c—glycated haemoglobin A1c; PA—frequency of physical activity; PA0: none; PA1: less than three times per month; PA2: one to two times per week; PA3: three to four times per week; PA4: more than five times per week. Mean ± SE of the data.

HbA1c values were also analyzed by gender, but no significant differences were found. When children who were physically active were grouped according to the frequency of physical activity, there was no significant difference between boys and girls (Figure 3).

## 4. Discussion

With increasing prevalence/incidence, childhood T1DM is a global challenge. Our findings are consistent with previous research showing that children with T1DM participate significantly less in sports than their healthy peers [43,50,54]. In line with previous studies [30,67], the most common reasons in the T1DM group of the present study for not exercising were lack of time (12.4% and 20.7% in the control and diabetic groups, respectively), fatigue (9% and 14.3% in the control and diabetic groups, respectively), and lack of motivation (10.1% and 10.7% in the control and diabetic groups, respectively). No association was found between BMI status and the physical activity of the children. Similarly, no significant association between physical activity and HbA1c levels was found in children with diabetes between those who did (7.65% ± 0.96) and those who did not (7.88% ± 0.96) engage in physical activity. A separate analysis of girls’ and boys’ responses showed no significant differences in either group in terms of physical activity rates, participation in physical education classes, and physiotherapy.

There was no significant difference between the responses of parents of physically active and inactive children with T1DM regarding the frequency of hypoglycaemic episodes. However, strikingly, a substantial proportion of parents—approximately two-thirds of respondents—reported that their child avoided participation in PE classes due to fear of hypoglycaemia. Giblin et al. reported in a similar study [53], consistent with the present findings, that 83% of parents of children with T1DM believe that the disease affects their child’s physical activity, and that fear of hypoglycaemia was among the most common fears. In the same study, the authors found that 47% of children with T1DM do not participate in physical education classes at school, while 21% do not participate in sports outside of school. Although the data obtained in the present study are somewhat different (11.7% do not participate in physical education classes and 42.5% do not participate in sports outside school), they show a similar trend, with a significant proportion of children not engaging in physical activity in the recommended amounts. Parents of diabetic children who exercised regularly believed that physical activity would improve their child’s diabetes and that their child could attend PE classes together with healthy children, compared to parents of diabetic children who were not physically inactive, where fewer agreed with the former statements. In these aspects, no significant differences were found between the responses of parents of boys compared to those who have girls. These findings underscore the importance of parental education as a critical component in promoting a healthy lifestyle among children with T1DM.

It is well documented that a healthy lifestyle should start in childhood [68], and regular physical activity is one of its pillars. In terms of physical activity habits, we need to distinguish children with chronic diseases such as T1DM, which is the case in our study, as it is not only about developing the habit of exercising, but there are other factors that influence the type and amount of physical activity, such as parental behaviour and motivation, disease awareness and control, and environmental attitudes.

The most important interventions that lead to an increase in physical activity in the long term are school and community health education, sports campaigns, community acceptance and support for chronic patients, community sensitization to healthy lifestyles, and healthy lifestyles shaped by positive parental role models [69,70].

Barriers to sport in children with diabetes can only be eliminated through proper patient education and patient experience. Each T1DM patient requires individual adjustment, personalized therapy, as they respond differently to different environmental influences. Thus, the more a person knows about their diabetes, the more courageous they are to exercise and the better their quality of life is. The attitude of the environment toward diabetes also matters a lot, especially for children of preschool and school age. The importance of appropriate education cannot be overemphasized, as it is the responsibility of the healthcare staff who care for T1DM patients [30].

In our region, education is provided partly in the hospital by specialists, nurses, dieticians, and possibly psychologists, and partly in patient clubs and diabetes camps. We believe that this continuous education and monitoring at follow-up visits is reflected in the fact that we did not find any differences between HgA1c levels of control and diabetic children. Furthermore, the proportion of diabetic children, who regularly exercise was albeit smaller than that of their healthy peers but nevertheless high. The latter could not be achieved without the education from dedicated and rested healthcare workers who tend these children in patient clubs, diabetes camps outside their working hours [71].

Due to time constraints, however, there is little opportunity to provide quality education to parents during follow-up visits. Therefore, experts—as in other countries—recommend close collaboration between families, school staff, and healthcare providers [72].

A limitation of the research is that only self-report questionnaires were used. The control group was not explicitly questioned about participation in exercise programs based on medical recommendation; therefore, their frequency of physical activity may not accurately reflect this. Perceiving children’s activity can present several challenges for parents, as movement is not always clearly observable and can be influenced by several factors. It would be important to collect and verify the data using objective methods. It would be beneficial to carry out the survey on sporting habits together with an assessment of the infrastructural environment, and on a larger sample. A more accurate picture would be obtained if the analysis could be carried out together with assessing the parents’ lifestyle habits.

These findings confirmed most of the initial hypotheses that fear of hypoglycaemia and parental concerns were associated with lower levels of physical activity in children with T1DM. The observed patterns were consistent across genders. However, the assumption that the severity of the disease would correlate with reduced physical activity and changes in BMI was not supported by the data. Overall, the results align with other studies in the field and highlight the need to address parental perceptions to encourage regular physical activity in this group of children.

## 5. Conclusions

The novelty of our study lies in the fact that it examined the sports habits of children living with T1DM in a disadvantaged region of Hungary and the parental attitudes underlying these habits. Based on our findings, children need to be encouraged to be more physically active; thus, motivation is a shared interest and responsibility of the child’s healthcare staff, teachers, and parents. T1DM patients need to be better educated about the role of exercise. They should be prepared to manage the metabolic changes induced by exercise. To increase the time spent exercising, it would be very helpful if children had more leisure time and motivation, and thus fatigue would be less of a barrier.

A useful initiative would be to establish and operate clubs where parents of children with T1DM can meet within the framework of classes and consultation hours at all healthcare institutions where these children receive check-ups. This initiative could serve as a platform where knowledgeable healthcare professionals could provide parents with information about the disease and its treatment, including recommendations on physical activity for children, answer parents’ questions and concerns, clarify misconceptions, and ease anxieties. In addition to professional guidance, parents could also share their experiences with each other regarding issues related to their children.

Future research should include an age-group analysis of the sporting habits of children with diabetes in relation to parental and school environments. It would also be important to explore what motivational tools could encourage children and their parents to engage in regular physical activity.

## Figures and Tables

**Table 1:** Variables and scores used in the survey.

Variables	Definition		
Demographic	Gender (boy, girl) Age (4–10 years, 11–14 years, 15–18 years) Place of residence (village, town, Debrecen, other county, Budapest) Education (elementary school, high school, vocational high school, technical college)		


Metabolic	BMI (<5th percentile for underweight, 5th–85th percentile for normal, 85th–95th percentile for overweight, ≥95th percentile for obese) HbA1c (<7% for good glycemic control, ≥7 for poor glycemic control)		

Clinical	Years of disease (<6 months, 6–12 months, >12 months) Treatment (MDIs, pump)		

Physical activity	Measured by IPAQ		
	Other daily activities, how often, what and where the child likes to play sports		
	Parents’ opinions on the relationship between physical activity and diabetes; the items used are as follows:		Likert scale from 1 to 5: 1 meaning “strongly disagree” and 5 “strongly agree”
	PA is important		
	The child likes vigorous PA		
	Certain sports are better		
	Same amount of PA as healthy peers		
	Diabetes is improved by PA		
	PA makes diabetes worse		
	PA is dangerous		
	Can cope with the requirements of PE lessons		
	Can participate fully in PE lessons		
	Daily PE lessons help		
	Daily PE has positively influenced their attitude		
	The transition from MDIs to insulin pumps has positively influenced their attitude		
Quality of life	Measured by PedsQL 3.0; the diabetes symptom items used are the following:		Scale from 0 to 4: 0 meaning “never” and 7 “almost always”
	I felt hungry		
	I felt thirsty		
	I have to go to the bathroom too often		
	I have stomach aches		
	I have headaches I go “low” I felt tired or fatigued I become shaky I become sweaty I have trouble sleeping I get irritable		

BMI: body mass index; HbA1c: glycated haemoglobin A1c; MDIs: multiple daily injections; IPAQ: International Physical Activity Questionnaire; PA: physical activity; PedsQL 3.0: Pediatric Quality of Life Inventory 3.0.

**Table 2:** Demographic characteristics of children with type 1 diabetes mellitus and healthy children.

	Children with T1DM (*n* = 140)	Healthy Children (*n* = 178)
Gender (%)		
Girls	48.2	55.1
Boys	51.8	44.9
Age groups (%)		
4–10	22.5	26.1
11–14	38.4	42.6
15–18	39.1	31.3
Mean ± SD	13.1 ± 3.2	12.6 ± 2.8
Education (%)		
Elementary school	54.6	63.6
High school	29.2	17.9
Vocational high school	10	11
Technical college	6.2	7.5
Place of residence (%)		
Village	26.1	22.6
Town	49.3	51.8
Debrecen	21.6	19
Other county	2.2	6.5
Budapest	0.7	0
BMI * (%)		
Underweight (<5th percentile)	6.7	7.9
Normal (5th–85th percentile)	64.2	55.8
Overweight (85th–95th percentile)	17.9	15.8
Obese (≥95th percentile)	11.2	20.6
Mean (kg/m^2^) ± SD	20.9 ± 6.5	21.4 ± 5.9
HbA1c (%)		
Mean ± SD	7.8 ± 1	–
Diabetes present (%)		
<6 months	2.9	–
6–12 months	5	–
>12 months	92.1	–
Mean (years) ± SD	6.2 ± 3.7	–
Insulin method (%)		
MDIs	53.6	–
Pump	46.4	–
Relatives have T1DM (%)	27.1	23.7
Physical activity (%)		
Yes	57.5	67.6
No	42.5	32.4
Frequency of PA (%)		
Over five times per week	22.6	14.3
Three to four times per week	36.9	36.1
One to two times per week	35.7	46.2
Three times per month or less	4.8	3.4

T1DM: type 1 diabetes mellitus; BMI: body mass index; HbA1c: glycated hemoglobin A1c; MDIs: multiple daily injections; PA: physical activity. * Data missing on BMI for six patients and 13 healthy children.

**Table 3:** Mean (SD) of parents’ opinions on physical activity in the study groups.

Statements	Children with T1DM, *n* = 140			Healthy Children, *n* = 178		
	Physical Activity	Physical Inactivity	*p*-Value ^a^	Physical Activity	Physical Inactivity	*p*-Value ^a^
Physical activity is important for children with diabetes.	4.68 (0.68)	4.23 (0.95)	***0.001***	–	–	–
The child likes vigorous physical activity.	3.44 (1.34)	2.29 (1.11)	***<0.001***	3.59 (1.17)	2.50 (1.11)	***<0.001***
Certain sports are better for children with diabetes.	3.68 (1.22)	3.39 (1.14)	0.124	–	–	–
Children with diabetes can do the same amount of physical activity as their healthy peers.	4.30 (0.94)	3.61 (1.33)	***0.002***	–	–	–
Diabetes is improved by physical activity.	4.39 (0.96)	3.82 (1.29)	***0.007***	–	–	–
Physical activity makes diabetes worse.	1.38 (0.89)	1.41 (0.81)	0.429	–	–	–
Physical activity is dangerous for children with diabetes.	1.76 (1.05)	1.81 (1.06)	0.786	–	–	–
My child feels that they can cope with the requirements of PE lessons.	4.09 (1.43)	3.91 (1.31)	0.127	4.31 (1.17)	3.76 (1.24)	***<0.001***
My child feels that they can participate fully in group sports activities in PE lessons.	4.58 (0.92)	4.11 (1.22)	***0.009***	4.67 (0.69)	3.86 (1.20)	***<0.001***
My child’s health has improved following the introduction of daily physical education.	3.75 (1.32)	3.02 (1.34)	***0.002***	3.85 (1.24)	3.22 (1.33)	***0.003***
My child’s attitude toward physical activity has been positively influenced by the introduction of daily PE.	3.82 (1.27)	2.83 (1.38)	***<0.001***	3.88 (1.27)	3.34 (1.26)	***0.008***
When my child switched from MDIs to an insulin pump, their attitude toward physical activity changed in a positive direction.	3.35 (1.53)	2.76 (1.52)	0.076	–	–	–

Significant *p*-values are indicated in bold and italic (*p* < 0.05). ^a^ Mann–Whitney–Wilcoxon test.

**Table 4:** The association between physical activity and diabetes symptoms.

Symptoms	Mean (SD)		*p*-Value ^a^
	Physical Activity	Physical Inactivity	
Hunger	2.61 (1.24)	2.80 (1.17)	0.332
Thirst	2.69 (1.22)	2.65 (1.26)	0.876
Frequent urination	2.18 (1.14)	2.14 (1.10)	0.930
Stomach pain	2.03 (1.04)	1.96 (1.10)	0.576
Headache	2.21 (1.03)	2.16 (0.98)	0.751
Blood sugar levels decrease	2.97 (0.82)	2.91 (0.79)	0.865
***Fatigue***	2.70 (1.16)	3.20 (1.16)	***0.015***
Tremor	1.93 (0.87)	1.89 (0.94)	0.695
Sweating	1.89 (1.08)	1.86 (0.94)	0.896
Bad sleep	1.83 (0.99)	1.91 (1.32)	0.627
Irritability	2.47 (1.11)	2.43 (1.33)	0.623

Significant *p*-values are indicated in bold and italic (*p* < 0.05). ^a^ Mann–Whitney–Wilcoxon test.

**Table 5:** Mean (SD) of parents’ opinions by gender on a scale of 1–5 in the control and diabetes groups.

Statement	Children with T1DM, *n* = 140			Healthy Children, *n* = 178		
	Girls	Boys	*p*-Value ^a^	Girls	Boys	*p*-Value ^a^
Physical activity is important for children with diabetes.	4.51 (0.73)	4.46 (0.93)	0.755	–	–	–
The child likes vigorous physical activity.	2.69 (1.33)	3.23 (1.35)	***0.024***	3.05 (1.32)	3.47 (1.14)	0.072
Certain sports are better for children with diabetes.	3.68 (1.18)	3.40 (1.21)	0.210	–	–	–
Children with diabetes can do the same amount of physical activity as their healthy peers.	4.08 (1.06)	3.94 (1.24)	0.719	–	–	–
Diabetes is improved by physical activity.	4.06 (1.14)	4.24 (1.14)	0.255	–	–	–
Physical activity makes diabetes worse.	1.45 (0.86)	1.23 (0.67)	0.086	–	–	–
Physical activity is dangerous for children with diabetes.	1.72 (0.97)	1.76 (1.06)	0.947	–	–	–
My child feels that they can cope with the requirements of PE lessons.	3.87 (1.48)	4.15 (1.27)	0.347	4.15 (1.21)	4.15 (1.23)	0.994
My child feels that they can participate fully in group sports activities in PE lessons.	4.34 (1.12)	4.48 (0.99)	0.450	4.36 (1.00)	4.63 (0.87)	0.382
My child’s health has improved following the introduction of daily physical education.	3.43 (1.42)	3.48 (1.31)	0.935	3.41 (1.34)	3.93 (1.17)	***0.012***
My child’s attitude toward physical activity has been positively influenced by the introduction of daily PE.	3.43 (1.38)	3.41 (1.44)	0.988	3.49 (1.32)	3.99 (1.18)	***0.016***
When my child switched from MDIs to an insulin pump, their attitude toward physical activity changed in a positive direction.	3.34 (1.44)	3.02 (1.62)	0.365	–	–	–

Significant *p*-values are indicated in bold and italic (*p* < 0.05). ^a^ Mann–Whitney–Wilcoxon test.

## Data Availability

The data are not publicly available due to privacy restrictions.
